# Involvement of the dorsal and ventral attention networks in visual attention span

**DOI:** 10.1002/hbm.25765

**Published:** 2022-01-04

**Authors:** Jing Zhao, Junkai Wang, Chen Huang, Peipeng Liang

**Affiliations:** ^1^ Key Laboratory of Learning and Cognition, School of Psychology Capital Normal University Beijing China; ^2^ Department of Psychology Tsinghua University Beijing China; ^3^ Department of Psychology Zhejiang University Hangzhou China

**Keywords:** a prospective visual 1‐back task, attention networks, neural mechanism, visual attention span

## Abstract

Visual attention span (VAS), which refers to the window size of multielement parallel processing in a short time, plays an important role in higher‐level cognition (e.g., reading) as required by encoding large amounts of information input. However, it is still a matter of debate about the underlying neural mechanism of VAS. In the present study, a modified visual 1‐back task was designed by using nonverbal stimuli and nonverbal responses, in which possible influences of target presence and position were considered to identify more pure VAS processing. A task‐driven functional magnetic resonance imaging (fMRI) experiment was then performed, and 30 healthy adults participated in this study. Results of confirmatory and exploratory analyses consistently revealed that both dorsal attention network (DAN) and ventral attention network (VAN) were significantly activated during this visual simultaneous processing. In particular, more significant activation in the left superior parietal lobule (LSPL), as compared to that in the bilateral inferior frontal gyrus (IFGs), suggested a greater involvement of DAN in VAS‐related processing in contrast to VAN. In addition, it was also found that the activation in temporoparietal junctions (TPJs) were suppressed during multielement processing only in the target‐absent condition. The current results suggested the recruitment of LSPL in covert attentional shifts and top‐down control of VAS resources distribution during the rapid visual simultaneous processing, as well as the involvement of bilateral IFGs (especially RIFG) in both VAS processing and inhibitory control. The present findings might bring some enlightenments for diagnosis of the atypicality of attentional disorders and reading difficulties.

## Graphical abstract

A prospective visual 1‐back task used during functional magnetic resonance imaging (fMRI) scanning were designed to examine the neural mechanism of visual attention span (VAS) which is a basic cognitive ability and plays an important role in higher‐level cognition (e.g., reading) as required by encoding large amounts of information input. Results of confirmatory and exploratory analyses consistently revealed greater involvement of dorsal attention network (DAN, e.g., left superior parietal lobule) as compared to ventral attention network (VAN, e.g., bilateral inferior frontal gyrus) in this visual simultaneous processing. The present findings bring some enlightenments for diagnosis of the atypicality of attentional disorders and reading difficulties.

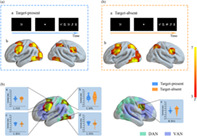

## 1 INTRODUCTION

Nowadays, the explosion of information makes the rapid simultaneous processing skill necessary for ensuring life quality and work efficiency (Liu, Jiang, Sun, & He, 2009; Wang & Zhang, 2021). In visual modality, this rapid processing capacity is closely associated with visual attention span (VAS), which refers to the window size of multielement processing in parallel in a short time frame (Bosse, Tainturier, & Valdois, 2007). VAS can be measured by whole/partial report tasks and relevant modified paradigms such as visual 1‐back and categorization tasks (Lallier, Acha, & Carreiras, 2016; Lobier, Zoubrinetzky, & Valdois, 2012). The multitrace memory model (Ans, Carbonnel, & Valdois, 1998) proposes an important role of VAS in the main methods of information input, that is, the reading procedure. As this model indicates, large VAS corresponds to an ability to extend the attentional window over the whole sequence of a word, which can further contribute to reading through the global lexical route (Stefanac et al., 2019). Most of the previous studies have focused on the relationship between VAS and reading‐related processing (Banfi et al., 2018; Bosse et al., 2007; Valdois et al., 2014; Valdois, Lassus‐Sangosse, Lallier, Moreaud, & Pisella, 2019). However, the VAS‐reading relationship has still been in debate in the current background of empirical studies. Accordingly, it is necessary to fundamentally explore the nature of VAS, so as to deepen our understanding about the underlying mechanisms of VAS in higher‐level cognition (e.g., reading) relating to the simultaneous encoding of a large amount of information input.

So far, the cognitive mechanism regarding VAS is still controversial. Some researchers indicated that VAS is one of the critical visuo‐spatial attention skills and mainly reflects top‐down attentional modulation (Valdois et al., 2019). One of the supporting evidences is that the window size of VAS is closely associated with the experience‐based distribution pattern of visual attentional resources (Lallier et al., 2016; Valdois et al., 2019). Position‐based analyses of behavioral data in the VAS‐related tasks showed an inverted “V” shape of the visual attentional distribution, which means the highest scores in the third position of the string and a decrease in performance with enlarging eccentricity (Tydgat & Grainger, 2009; Ziegler, Pech‐Georgel, Dufau, & Grainger, 2010). However, some other researchers indicated that VAS is not limited to the cognitive processing of loading visual attentional resources across space, which can be divided into two types of attentional subcomponents, that is, the bottom‐up stimulus‐driven attention including visual short‐term memory storage and perceptual processing speed, and the top‐down attentional control including spatial attentional weight and distractor inhibition (Bogon et al., 2014; Bundesen, 1990; Dubois et al., 2010; Stefanac et al., 2019). Then, what is the underlying mechanism about VAS? Whether it is only related to the top‐down attentional control (i.e., distribution of visual attention resources) or it requires the conjoint involvement of both bottom‐up and top‐down attentional processes? The current study attempts to address these issues.

### 1.1 Dissociation of neural mechanisms relating to the bottom‐up and top‐down attentional processes

Bottom‐up and top‐down attentional processes have (partially) separate mechanisms in the neural aspect (Corbetta & Shulman, 2002; Weissman & Prado, 2012). Especially, the top‐down attentional control (i.e., endogenous attention) mainly relies on the dorsal attention network (DAN) to orient visuospatial attention and to maintain endogenous signals relating to the current task goals, which are classically implicated with the function in brain regions of bilateral frontal eye fields (FEF) and bilateral posterior parietal cortex (Berndt et al., 2019; Corbetta & Shulman, 2002); meanwhile, dorsolateral prefrontal cortex and portions of dorsal anterior cingulate cortex have also been reported to be recruited in the related control processes (Orr & Weissman, 2009; Weissman & Prado, 2012). In contrast, the stimulus‐driven attention (i.e., exogenous attention) remarkably activates the ventral attention network (VAN) to reorient the visuospatial attention, which mainly evokes brain activities in inferior frontal gyrus (IFG), temporoparietal junctions (TPJs), especially in the right hemisphere (Corbetta & Shulman, 2002). Therefore, neuroimaging researches on the two attention networks functioning in bottom‐up and top‐down attentional processes could contribute to examining the underlying mechanism of VAS.

### 1.2 Previous neuroimaging studies on visual attention span

#### 1.2.1 Research based on traditional VAS tasks with verbal response and verbal stimuli

Peyrin, Lallier, and Valdois (2008) firstly conducted a functional magnetic resonance imaging (fMRI) study to explore the neural mechanism of VAS with a 5‐letter global report task, which was similar to the paradigm used in the behavioral studies except that the response was changed to silent report instead of oral report. As compared to the experimental condition of the silent report task, a silent counting task was used as a control condition, in which participants were required to count silently from 1 to 5 when they saw a five‐symbol string. Comparisons in the neural activities between the experimental and control conditions showed significant activation in regions belonging to both VAN (e.g., left angular gyrus) and DAN (e.g., left superior parietal lobule). However, this global report task used silent reports as responses, measuring aspects, which were implicated with linguistic‐related processing such as visual‐to‐semantic mapping and visual‐to‐phonological transfer (Wang et al., 2015). It has been reported that the interaction among visual, phonological, and semantic information during silent reading would also evoke the brain activations in temporoparietal areas, which partially overlap with VAN and DAN (Wang et al., 2015).

#### 1.2.2 Research based on modified VAS tasks with nonverbal response and verbal stimuli

In the following studies, researchers designed a flanked letter categorization task and a perceptual matching task to measure VAS capacity while minimizing oral report and linguistic processing (Peyrin et al., 2012; Peyrin, Démonet, N’Guyen‐Morel, Le Bas, & Valdois, 2011; Valdois et al., 2014; Valdois, Peyrin, & Baciu, 2009). In the flanked letter categorization task, the stimuli were a pair of letters, and participants were required to judge whether the two stimuli were the same or not. In particular, the target stimulus was flanked by other two letters in the experimental condition while it was presented alone in the control condition. As to the perceptual matching task, participants were required to judge whether two successively presented 5‐letter strings were identical or not by pressing the corresponding keys. As compared to the control condition or baseline, adult and child participants greatly activated superior parietal lobule (the classical DAN regions) as well as supramarginal gyrus and inferior frontal gyrus (belonging to VAN) in the experimental condition (Peyrin et al., 2011; Reilhac, Peyrin, Démonet, & Valdois, 2013; Valdois et al., 2014). The above findings revealed significant recruitments of dorsal and ventral attention networks tapping VAS‐related skills after excluding the possible interruption from the verbal responses. Yet, it should be noted that the stimuli in the above studies are still letters. Processing verbal stimuli has been found to evoke the regions of VAN such as supramarginal gyrus and angular gyrus (Ekstrand, Neudorf, Gould, Mickleborough, & Borowsky, 2019; Richards et al., 2015). Therefore, whether the activation in regions belonging to VAN reflects the cognitive function regarding VAS or the linguistic processing of verbal stimuli requires to be further examined.

#### 1.2.3 Research based on modified VAS tasks with nonverbal response and nonverbal stimuli

Later studies made efforts on designing more advanced paradigms tapping putative VAS capacity, such as the novel categorization task with nonverbal stimuli designed by Lobier et al. (2012); Lobier, Peyrin, Pichat, Le Bas, and Valdois (2014). In this task, participants were asked to respond the number of special characters in a string by pressing relevant buttons in the multielement condition, and to judge whether or not the single stimulus belonged to one special type by pressing the corresponding buttons in the single‐element condition. The authors found that the comparisons in brain activation between multiple‐element and single‐element conditions showed significant differences in bilateral superior parietal lobules functioning in orienting visuospatial attention (Lobier et al., 2012, 2014). This finding suggested a remarkable involvement of DAN but not VAN in a VAS task without the possible influence of verbal stimuli. However, the above modified VAS task (i.e., visual categorization task) required the participants to count the number of one type of items and to hold the relevant stimuli online in the short‐term memory, and the counting procedure would be implicated with neural activities in and around the intraparietal sulcus (Park, Hebrank, Polk, & Park, 2012). Thus, it is necessary to recheck whether the superior parietal activities reported in the studies of Lobier et al. (2012, 2014) are due to VAS itself or number counting. In the future research, designing an fMRI paradigm with both nonverbal stimuli and nonverbal responses while excluding the involvement of cognitive requirements other than visual attention would allow for the assessment of more putative neural correlates of VAS.

### 1.3 Aims of the present study

Previous literature has explored the neural mechanism about VAS, however, it is still in debate: Whether both VAN and DAN or only DAN underpins visual attention span? Whether the involvement of VAN is special to VAS skill (especially for the stimulus‐driven attention subcomponents) when the linguistic‐related factors are controlled?

In order to address these issues, *the initial aim of the current study* is to develop a prospective visual 1‐back task based on a partial report task (i.e., one of the traditional VAS tasks) to adapt to the neuroimaging study on VAS‐related processing. In traditional VAS tasks, the procedure within one trial, that a cue follows a string, confuses the string identification (i.e., VAS processing) with the cue processing, in which it is difficult to measure putative VAS‐related processing during neural scanning. Yet in the present modified task, a cue is presented before a series of string stimuli, and participants are required to make a response to each string, which makes the cognitive processes within each trial mainly involve the string processing.

*The second aim of the present study* is to examine the neural correlates of VAS through the prospective visual 1‐back task, with further investigating the neural mechanism underlying the attentional distribution of VAS resources by comparing different conditions concerning target positions.

*The third aim of the present study* is to pinpoint VAS‐related brain activation by contrasting multielement processing to single‐element processing separately within target‐present and target‐absent conditions. In most of the neuroimaging studies on VAS, the neural response to multielement processing is contrasted with the response to single‐element processing without taking the influence of conditions regarding target presence into account. Particularly, response inhibition is greatly involved in the target‐absent condition, and it has been suggested that some regions belonging to the two attention networks (e.g., intraparietal sulcus and TPJs) are implicated in the inhibition process (Kolodny et al., 2017; Pollmann et al., 2003; Wei, Müller, Pollmann, & Zhou, 2009). It thus transpires that these regions are not triggered exclusively by the mere multielement processing, instead, it may engage in the response inhibition. Meanwhile, cue‐induced orienting in the present VAS task might be implicated in attentional selection besides the rapid visual simultaneous processing, which could also be specially reflected by the comparisons between the target‐present (selected) and target‐absent (nonselected) conditions. It could be proposed that, if one brain region is similarly activated in both conditions concerning target presence, then this region would be regarded as a candidate of neural correlates of VAS. Otherwise, if one brain region is greatly activated in the target‐absent condition in which participants are required to inhibit the activated presentations of all the items in a string, while less activated or not evoked in the target‐present condition, then this region is probably considered to be responsible for the inhibition process; if one brain region is greatly activated in the target‐present condition as compared to the target‐absent condition, then this region may be regarded to mainly function as attentional selection.

## 2 METHODS

### 2.1 Participants

Thirty healthy adults (17 males and 13 females, mean age: 22.07 ± 3.10 years) were recruited in the present study and were paid for their participation. The datasets of three participants (3 males) were excluded from further data analyses because their accuracy in the present VAS task was below 50%. All participants were right‐handed and had normal or corrected‐to‐normal vision without ophthalmologic or neurological abnormalities. Written informed consent was obtained before the formal experiment. The study was carried out in accordance with the relevant guidelines and regulations. The research project was approved by the Research Ethics Committee of the School of Psychology, Capital Normal University.

### 2.2 Visual stimuli

Ten symbols designed on the basis of previous literature (Zhao et al., 2019; Zhao, Liu, Liu, & Huang, 2018) were used as nonverbal stimuli in the present study, of which the visual complexity and visual familiarity were evaluated by another 35 university students (16 males and 19 females, mean age: 23.19 ± 1.68 years) who did not take part in the formal study. A five‐point rating scale was adopted during the evaluation, in which 1 point represents “The symbol is extremely simple”/ “The symbol is extremely familiar” and 5 points represent “The symbol is extremely complex”/ “The symbol is extremely strange”. Results showed that the average rating scores of visual complexity and visual familiarity of these symbols were 2.30 ± 0.15 and 2.36 ± 0.14, respectively, revealing mid‐level degrees in their visual complexity and familiarity. The visual complexity and familiarity of any two of the 10 symbols did not significantly differ from each other (*p* > .1, Bonferroni corrected). Detailed information about these symbols and their properties were shown in Table 1. Eighty‐four five‐symbol strings with the visual angle of 7.9° × 0.8° and a center‐to‐center distance between each adjacent item of 1.7° at a viewing distance of 50 cm were generated, in which no symbol was repeated in one string. In detail, 4 strings were for the practice section and 80 strings were for the formal experiment in the multiple‐element condition.

**TABLE 1:** Rating scores of visual complexity and visual familiarity for 10 symbols

Symbols	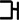	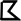	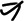	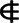	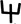	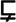	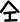	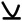	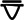	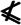
Visual complexity	2.44 (.11)	2.25 (.13)	2.13 (.23)	2.19 (.11)	2.63 (.27)	2.30 (.13)	2.13 (.11)	2.69 (.28)	2.00 (.10)	2.31 (.13)
Visual familiarity	2.94 (.21)	2.38 (.16)	2.25 (.19)	2.25 (.17)	3.00 (.19)	2.31 (.14)	2.06 (.19)	2.69 (.13)	1.94 (.18)	1.81 (.18)

*Note*: Standardized deviations were in the parentheses.

### 2.3 Visual attention tasks

In order to adapt to the neuroimaging study, modified visual 1‐back tasks were carried out in multiple‐element and single‐element conditions.

**FIGURE 1:**
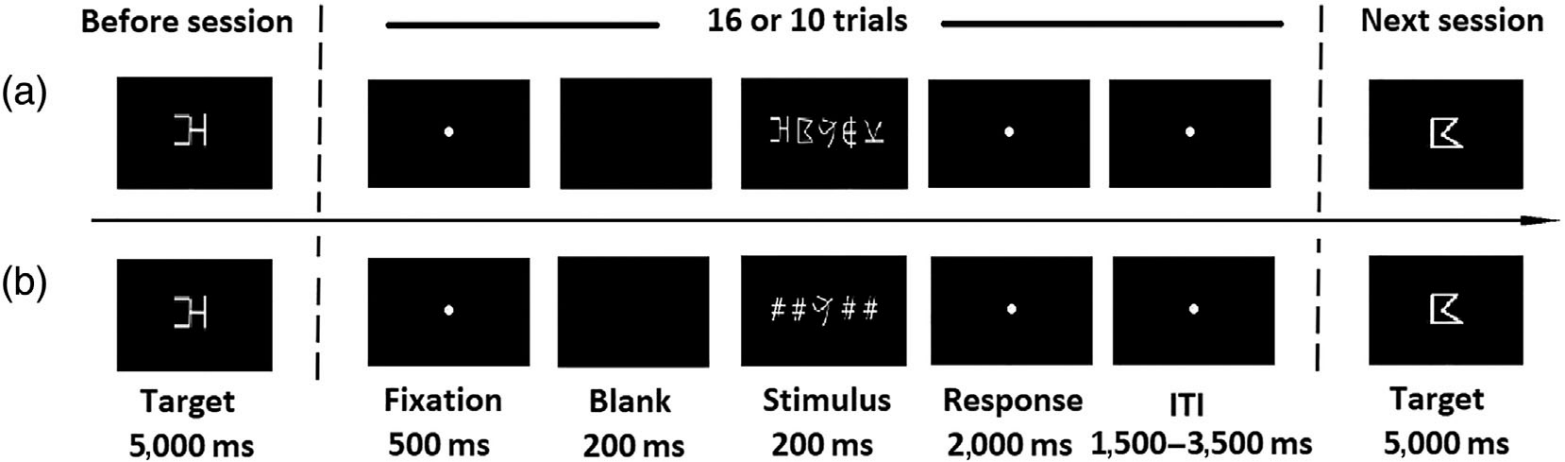
Presentation formats for multielement (a) and single‐element (b) identification tasks. Each session contains 16 trials for the multielement condition and 10 trials for the single‐element condition

*For the multiple‐element identification task*, there were 5 sessions with 16 trials in each session. A graphical description of this task is presented in Figure 1a. A target (i.e., a cue) appeared before each session for 5,000 ms. Participants were required to remember this symbol. Then 16 successive trials with string stimuli were presented, which included 8 target‐absent trials and 8 target‐present trials (4 trials for target presenting at the third position of a string, 2 trials at the first position, and 2 trials at the fifth position). Within each trial, a fixation dot first appeared at the center of the screen for 500 ms, which was followed by a 200‐ms blank premask, and then a five‐symbol string was presented at the center of the screen for 200 ms; after that, a fixation dot appeared in the screen center, and participants were asked to judge whether the five‐symbol string contained the target or not by pressing relevant keys within a time window of 2000 ms. After this time window, there was an intertrial interval with 2,500 ms on average (ITI = 1,500 ms, 2000 ms, 2,500 ms, 3,000 ms, 3,500 ms). This block consisted of 80 trials, and lasted about 9.73 minutes.

*For the single‐element identification task* (Figure 1b), the relevant performance was regarded as the baseline during neuroimaging data analyses. The stimuli were the 10 symbols mentioned above. There were totally 50 trials lasting about 6.23 minutes, which were equally divided into 5 sessions. The presentation procedure and property settings in this task were generally in line with those of the multielement condition, except that the stimuli comprised one symbol instead of five symbols.

The visual tasks were programmed by E‐Prime 1.1 software (E‐Prime Psychology Software Tools, Inc. Pittsburgh, USA). Synchronization between scanner and paradigm was ensured by a trigger pulse sent from the scanner to the computer on which E‐Prime was running. The paradigm was presented by a video projector (Epson EMP 8200), a projection screen situated behind the magnet and a surface mirror centered above the participant’s eyes. An MRI‐compatible response box was used to collect participant responses. Response accuracy and reaction time were recorded in the multiple‐ and single‐element identification tasks. We further computed d‐prime (d’) values on the basis of accuracy. The d’ values which were suggested to be a bias‐free estimate of task sensitivity (Lallier et al., 2016) and reaction times in the visual attention tasks were put into the following analyses.

### 2.4 Experimental procedure

**FIGURE 2:**
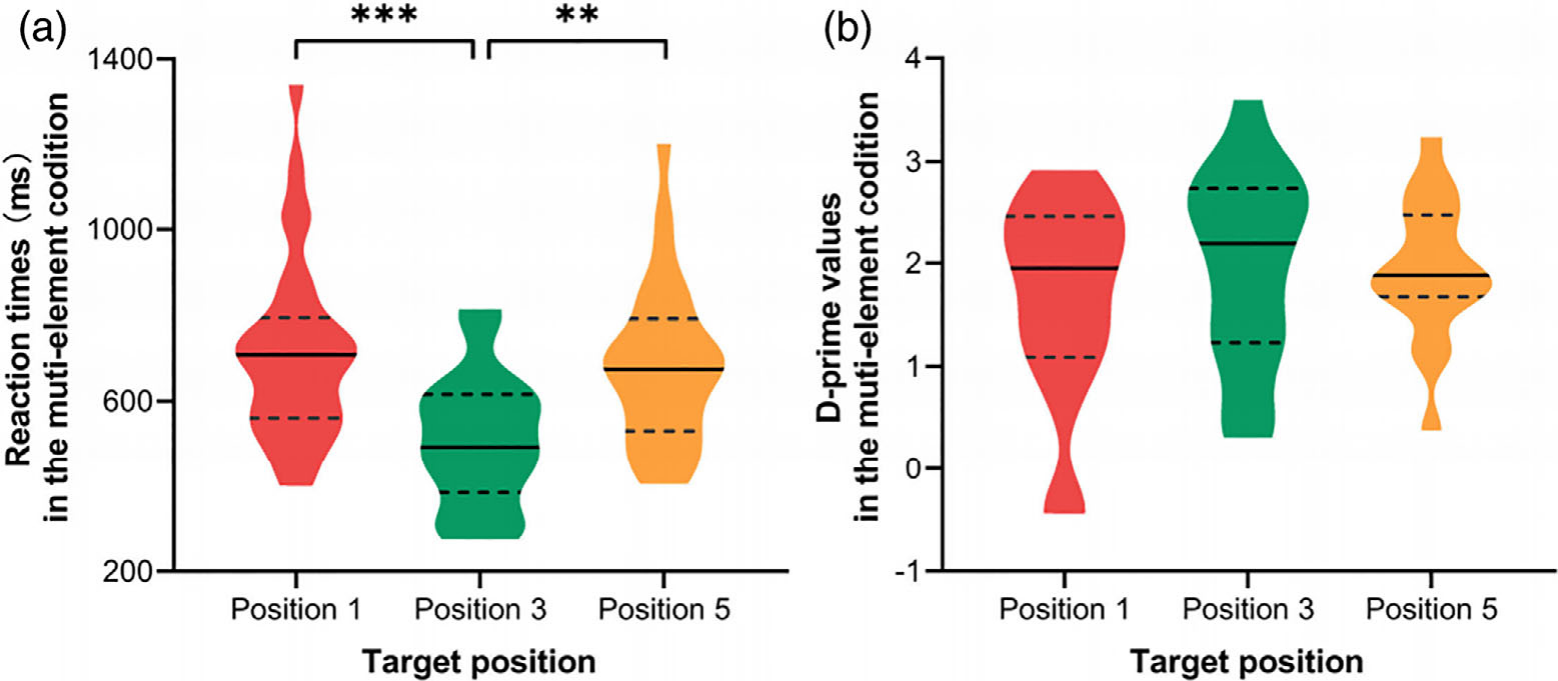
Position‐based analyses on correct reaction times (a) and d’ values (b) of the visual 1‐back task with multiple elements while controlling the corresponding responses in single‐element condition. Error bars represent standardized deviations. ***, *p* < .001; **, *p* < .01

**FIGURE 3:**
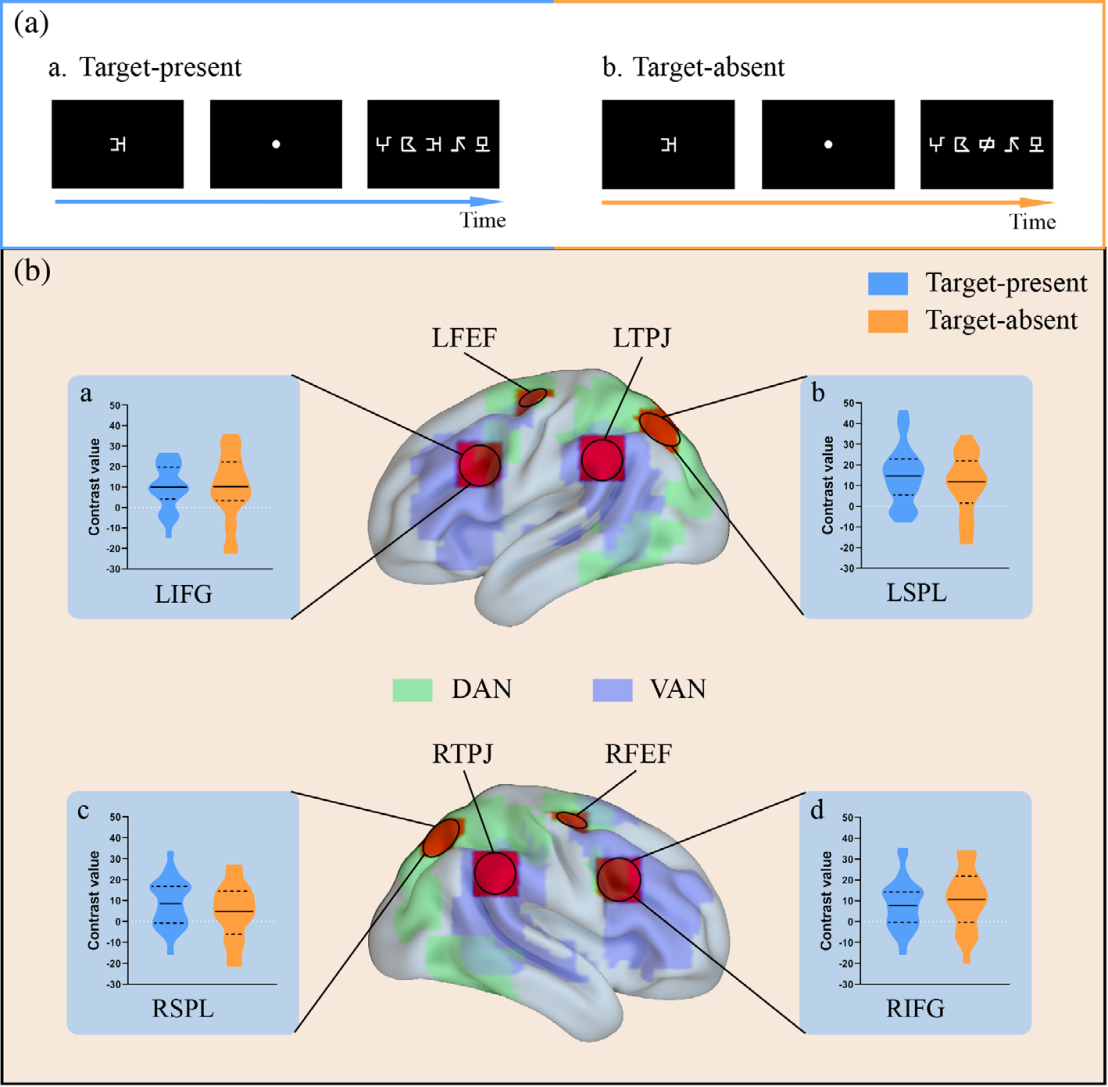
Comparisons of brain activations between the two conditions of target presence in ROIs extracted by the coordinates from the study of Fox, Corbetta, Snyder, Vincent, and Raichle (2006). A, examples of a target‐present trial (a) and a target‐absent trial (b). B, selected ROIs in the confirmatory analysis and violin plots about the comparisons of relevant contrast values between the target‐present (blue violin plots) and target‐absent (orange violin plots) conditions. DAN, dorsal attention network, the green areas over the brain; VAN, ventral attention network, the purple areas over the brain. The base maps of dorsal and ventral attention networks were depicted by analyzing the synchronous acquisition of resting‐state fMRI data. L, left; R, right; FEF, frontal eye field; IFG, inferior frontal gyrus; SPL, superior parietal lobule; TPJ, temporoparietal junction

Before attending the fMRI scan, participants conducted the experimental task outside the scanner to get familiar with the tasks. During the fMRI scan, participants performed 4 runs: the first run for an anatomical T1‐weighted scan; the second run for a functional resting‐state scan, the data of which was used to define dorsal and ventral attention networks, especially for the present study (as shown in Figures 2, 3); the third run for the single‐element condition; and the fourth run for the multielement condition. The total duration in the scanner was approximately 30 minutes for each participant.

### 2.5 Imaging acquisition

Hemodynamic responses were acquired on a 3‐Tesla Siemens Trio Magnetic Resonance Imaging (MRI) system in Peking University. Participants were instructed to keep their heads and bodies still inside the scanner, and their heads were aligned to the center of the magnetic field. For each participant, three‐dimensional anatomical images with high resolution were acquired using a Siemens magnetization‐prepared rapid acquisition gradient echo (MPRAGE) sequence (192 contiguous sagittal slices thickness = 1 mm, repetition time = 2,530 ms, echo time = 2.98 ms, field of view = 256 mm, flip angle = 7°, voxel size = 0.5 mm × 0.5 mm × 1 mm). For the functional imaging study, a blood oxygen level‐dependent (BOLD)‐sensitive gradient echo‐plane imaging (EPI) sequence was acquired. The following scan parameters were used: repetition time = 2000 ms, echo time = 30 ms, flip angle = 90°, field of view = 224 mm, number of slices = 62, slice thickness = 2 mm, voxel size = 2 mm × 2 mm × 2 mm.

### 2.6 Data analyses

#### 2.6.1 Behavioral data analyses

Firstly, the absolute values of correct reaction times in the visual attention tasks, which were three standard deviations above the mean, were excluded. The d’ values and remaining reaction times at the first, third, and fifth positions in the multielement condition were separately submitted to one‐way ANCOVAs with positions within a string (1, 3, 5) as the within‐subject factor and with corresponding responses in the single‐element condition as covariate, so as to compare the relevant VAS capacity across target positions with decreasing the influence of efficiency in single‐element processing. Post hoc analyses and multigroup comparisons were performed with Bonferroni correction.

#### 2.6.2 Preprocessing and analyses of task‐fMRI data

##### Preprocessing the task‐fMRI data

Data preprocessing and analyses were performed using SPM12 (Statistical Parametric Mapping) (http://www.fil.ion.ucl.ac.uk/spm/). In order to stabilize the magnetic field, each functional run started with 3 dummy scans that were removed from analyses. In the preprocessing, each individual dataset was corrected for slice timing and motion, spatially normalized into the standard Montreal Neurological Institute (MNI) template space, resliced to 3 mm × 3 mm × 3 mm voxels, and smoothed with an isotropic Gaussian kernel of 6 mm full‐width half‐maximum (FWHM).

##### Statistical analysis of task‐fMRI data

Statistical analysis was employed on the smoothed data. A first‐level general linear model analysis was performed for each participant. In order to pinpoint the VAS‐related brain activities, we took the possible influence of target presence into account. Consequently, four conditions of interests were set, including target‐absent trials in the single‐element identification (Condition 1) and multielement identification (Condition 2) tasks, and target‐present trials in the single‐element identification (Condition 3) and multielement identification (Condition 4, that is, especially for the target appearing at the middle position of a string to balance the target position between single‐ and multielement levels) tasks. Two types of contrasts were computed to examine the VAS‐related brain activation, that is, the contrasts between Condition 2 and 1, and the contrasts between Condition 4 and 3. Moreover, we also attempted to examine the position‐based neural correlates regarding VAS through the contrasts of the target‐present trials in the multielement session between the center position and noncenter position of a string.

In order to examine whether the neural correlates in respect to VAS involved regions in DAN or that in VAN or both networks, we conducted analyses based on regions of interest (ROIs). There were two kinds of methods to get the seeds for extracting ROIs: (1) Confirmatory analysis based on the seeds extracted from previous literature. According to Fox et al. (2006), DAN‐related seeds included bilateral FEFs and SPLs, meanwhile the seeds regarding VAN included bilateral IFGs and TPJs, relevant MNI coordinates are shown in Table 2. (2) Exploratory analysis based on the whole brain activation. Parameter estimates from the above contrasts in each participant model were entered into random‐effect analysis in the group using one‐sample *t* test. All reported areas of the whole brain activation were significant using FDR *p* < .05, with a cluster size greater than 30 voxels. The overlapping areas related to the multi‐ versus single‐element processing between the target‐absent and target‐present conditions were selected to be the specific set of VAS‐related ROIs. The seeds for these ROIs were identified by the average coordinates of the voxels with the local maximum t values in the two conditions regarding target presence.

**TABLE 2:** MNI coordinates for each of the regions of interest

Regions of interest	MNI coordinates			BA
	x	y	z	
*Confirmatory analysis*				
L FEF	−25	−12	55	6
R FEF	28	−10	53	6
L SPL	−22	−68	46	7
R SPL	20	−67	51	7
R IFG	47	14	32	9
L IFG	−47	14	32	9
R TPJ	57	−43	34	40
L TPJ	−57	−43	34	40
*Exploratory analysis*				
L IFG	−45	−2	30	9
R IFG	48	8	33	9
L FEF	−27	0	54	6
L SPL	−24	−60	50	7
L ITG	−44	−65	−7	19

*Abbreviations*: BA, Brodmann area; FEF, frontal eye field; IFG, inferior frontal gyrus; ITG, inferior temporal gyrus; L, left hemisphere; MFG, middle frontal gyrus; R, right hemisphere; SPL, superior parietal lobule; TPJ, temporoparietal junction.

By using in‐house Matlab code (Mathworks, Natick, MA, USA) calling functions in Marsbar 0.42 (Brett, Anton, Valabregue, & Poline, 2002), ROIs were extracted with a 3‐mm radius sphere centered at the coordinates of the above seeds separately in confirmatory and exploratory analyses. Contrast values between multiple‐element processing and single‐element processing in both of the target‐absent and target‐present conditions for each ROI were computed, and so were the contrast values between the target appearing in the center position of a string and that in the noncenter position. These contrast values between target‐present conditions and between different target positions were separately compared by paired *t* test. If an ROI was significantly activated to a similar extent in the two conditions of target presence, then this region was regarded to be closely related to VAS. In terms of the neural mechanism of VAS, if a significant difference in activation was observed between different target positions in one ROI, then this region might be responsible for attentional distribution during the rapid simultaneous processing.

## 3 RESULTS

### 3.1 Behavioral results

*Reaction times*. ANCOVA on the reaction time for each target position in the multielement condition showed a significant main effect of position [*F*(2, 48) = 5.36, *p* = .008, η ^2^ = .18] after controlling the possible influence from single‐element processing (Figure 2a). Multiple comparisons showed that the reaction time in the third position was shorter than that at the first and fifth positions (*p*s < .01) while no significant difference in reaction times between the first and fifth positions (*p* = .20).

*d’ values*. Results of ANCOVA showed that there were no significant differences in d’ values across target positions [*F*(2, 22) = 1.47, *p* = .25, η ^2^ = .12] (Figure 2b).

### 3.2 Task‐fMRI results

#### 3.2.1 Confirmatory analysis based on the seeds in previous literature

Firstly, for all the ROIs extracted based on the study of Fox et al. (2006), the contrast values between multiple‐ and single‐ element processing were submitted to one‐sample *t* test separately in target‐absent and target‐present conditions, so as to examine whether this region was significantly activated. Results (Figure 3) showed significant activations in IFGs, SPLs, and TPJs in bilateral hemispheres, in which bilateral TPJs were only negatively activated (i.e., greater activation in multielement processing than that in the single‐element processing) in the target‐absent condition but not in the target‐present condition. Bilateral IFGs and SPLs exhibited the significant activations in both conditions regarding target presence, and results of further paired‐sample *t* test showed that there were no significant differences between the target‐absent and target‐present conditions (LSPL: *t*_*26*_ = 1.60, *p* = .12; RSPL: *t*_*26*_ = 1.31, *p* = .20; LIFG: *t*_*26*_ = .06, *p* = .95; RIFG: *t*_*26*_ = .97, *p* = .34).

Moreover, we examined the possible laterality effects for brain activations in bilateral IFGs and SPLs via the paired‐sample *t* test. Results (see Figure S1 in the Supporting Information) showed greater activations in LSPL than that in RSPL for both conditions regarding the target presence (Target‐present condition: *t*_*26*_ = 2.43, *p* = .02; Target‐absent condition: *t*_*26*_ = 2.62, *p* = .02); while no significant lateralization was observed in the inferior frontal activities (Target‐present condition: *t*_*26*_ = .89, *p* = .38; Target‐absent condition: *t*_*26*_ = .36, *p* = .72).

#### 3.2.2 Exploratory analysis based on the whole brain activations

**FIGURE 4:**
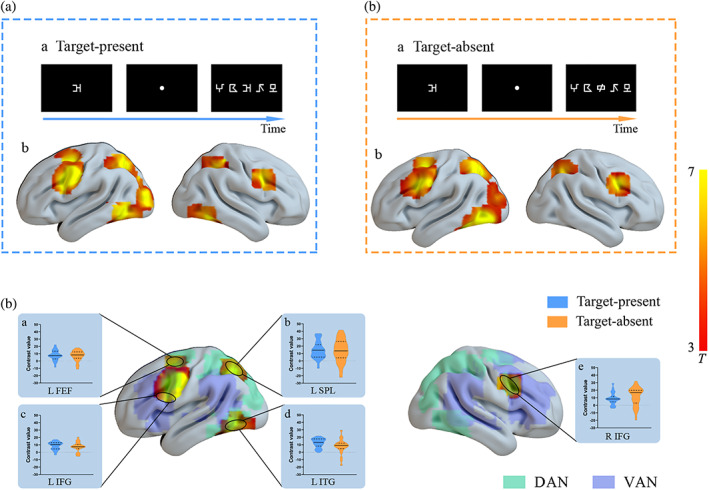
Brain regions showing activations as results of the whole‐brain paired‐sample *t* test comparisons in different contrasts. A, examples of a target‐present trial (a) and group activations by contrasting multielement processing versus single‐element processing in the target‐present condition (b). B, examples of a target‐absent trial (a) and group activation by contrasting multielement processing versus single‐element processing in the target‐absent condition (b). C, group activation in overlapping regions between the two conditions of target presence and violin plots in each condition for each ROI. DAN, dorsal attention network, the green areas over the brain; VAN, ventral attention network, the purple areas over the brain. The base maps of dorsal and ventral attention networks were depicted by analyzing the synchronous acquisition of resting‐state fMRI data. LFEF, left frontal eye field; LIFG, left inferior frontal gyrus; RIFG, right inferior frontal gyrus; LSPL, left superior parietal lobule; LITG, left inferior temporal gyrus. All reported areas of activations were significant using FDR *p* < .05, cluster size >30 voxels

By using the methods stated in the 2.6.2 section, the overlapping areas relating to the multi‐ versus single‐element processing between the two conditions of target presence included LIFG [−45, −2, 30], RIFG [48, 8, 33], LFEF[−27, 0, 54], LSPL [−24, −60, 50], and LITG [−44, −65, −7]. As shown in Figure 4, paired‐sample *t* test showed nonsignificant differences in activations between target‐absent and target‐present conditions in bilateral IFGs (LIFG: *t*_*26*_ = 1.12, *p* = .27; RIFG: *t*_*26*_ = 1.03, *p* = .31), LFEF (*t*_*26*_ = .40, *p* = .70), or LSPL (*t*_*26*_ = .39, *p* = .70), while LITG was more greatly activated in target‐present condition as compared to the target‐absent condition (*t*_*26*_ = 2.33, *p* = .03).

Furthermore, inferior frontal activities between left and right hemispheres were compared by a paired‐sample *t* test to examine the possible laterality effect. Results showed a greater activation in RIFG than that in LIFG in the target‐absent condition (*t*_*26*_ = 2.23, *p* = .04), while no lateralized difference in the target‐present condition (*t*_*26*_ = .17, *p* = .87).

#### 3.2.3 A combination of confirmatory and exploratory analyses

Results of these two types of analyses consistently exhibited significant and stable activations in bilateral IFGs and LSPL relating to visual simultaneous processing. There were no significant differences in brain activities between target‐absent and target‐present conditions for any of these three ROIs (*p*s > .05), no matter in confirmatory or in exploratory analysis (Table 3). Furthermore, we computed and compared the average activations of both conditions regarding target presence across these ROIs. As shown in Table 3, results of repeated‐measure ANOVA showed a significant main effect of regions in exploratory analysis [*F*(2, 52) = 4.76, *p* = .013, *η* ^*2*^ = .16]. Post‐hoc analysis exhibited that the activation in LSPL was more significant than that in bilateral IFGs (Bonferroni‐corrected *p*s < .05), while there was no any other significant effect.

**TABLE 3:** Means and standardized deviations of contrast values in each comparison and condition for three critical ROIs including LSPL and bilateral IFGs

Condition	LSPL	LIFG	RIFG
*Confirmatory analysis*			
Target‐absent condition	10.55 (14.39)	9.78 (15.85)	10.69 (14.09)
Target‐present condition	14.71 (13.98)	9.99 (10.95)	8.10 (11.82)
Average of both conditions regarding target presence	12.63 (12.47)	10.71 (9.62)	9.47 (8.29)
Position effect (outer vs center condition)	6.49 (9.41)	3.80 (11.14)	2.69 (8.67)
*Exploratory analysis*			
Target‐absent condition	13.64 (15.64)	7.45 (6.25)	11.35 (12.40)
Target‐present condition	14.68 (11.58)	9.00 (5.23)	8.74 (7.71)
Average of both conditions regarding target presence	14.16 (11.90)	8.23 (4.51)	10.05 (7.93)
Position effect (outer vs center condition)	5.85 (9.30)	0.27 (6.05)	1.74 (9.92)

*Note*: Standardized deviations were in the parentheses. LSPL, left superior parietal lobule; LIFG, left inferior frontal gyrus; RIFG, right inferior frontal gyrus.

#### 3.2.4 Position‐based analysis

Behavioral results revealed a fixation advantage in attentional distribution regarding VAS resources. In order to further investigate, it is neural mechanism, brain activations with respect to the effect of target position were examined. Because participants' responses to the targets presented at the first and fifth positions of a string were similar in the behavioral results, we combined these two levels together and regarded them as the “outer” position of a string in the following analyses. Contrast values between target appearing in the outer position and that in the middle position were computed in the ROIs stably reflecting the neural mechanism of VAS, that is, bilateral IFGs and LSPL. For these ROIs in both confirmatory and exploratory analyses, results (Table 3) showed that multielement processing in the outer position evoked greater brain activities in LSPL as compared to that in the middle position (*p*s < .05), while no significant difference between positions was observed in other ROIs (*p*s > .05).

## 4 DISCUSSION

The present study explored the neural mechanism of VAS by a trial‐by‐trial modified visual 1‐back task with nonverbal stimuli and nonverbal responses to minimize the possible influence of linguistic processing. Meanwhile, we took the modulation of target presence into account during fMRI data analyses, and compared the multielement processing to the single‐element processing separately within the target‐absent and target‐present conditions. In the combination of confirmatory and exploratory analyses, we consistently found that visual simultaneous processing was closely related to the activations in regions belonging to VAN (i.e., bilateral IFGs) and DAN (i.e., LSPL). Especially, LSPL showed greater activation than bilateral IFGs, suggesting more involvement of DAN during VAS‐related processing. Moreover, significant suppressions on bilateral TPJs were observed during multielement processing only in the target‐absent condition, revealing the close relationship between TPJs and inhibition processes; meanwhile, LITG showed greater activation in target‐present condition than that in target‐absent condition, suggesting its possible role in general selection of attention.

### 4.1 A special role of left superior parietal activities in VAS‐related processing

LSPL was found to be significantly activated in the target‐absent and target‐present conditions to similar extents, indicating that this brain region may be critical during visual simultaneous processing. Moreover, LSPL exhibited greater activation than RSPL, revealing a left lateralization in superior parietal activities which was inconsistent with previous literature reporting that VAS corresponded to brain activations in bilateral SPLs (Lobier et al., 2012, 2014; Peyrin et al., 2012; Valdois et al., 2019) with showing a right‐lateralized trend (Peyrin et al., 2011; Reilhac et al., 2013; Valdois et al., 2014). These previous studies on VAS (e.g., Peyrin et al., 2011, 2012; Reilhac et al., 2013) always reported the brain activations regarding the comparisons between the multielement and the single‐element conditions without considering the possible influence of the target item’s position in a string, in which overt attentional shifts might be unintentionally implicated, even though the 200‐ms duration for presenting the string stimulus reduced the possibility of overt attentional shifts during the string processing (Carrasco & Hanning, 2020; Lallier, Carreiras, Tainturier, Savill, & Thierry, 2013; Talcott & Gaspelin, 2020). By contrast, we took the target presence into account during analyzing the neuroimaging data, and examined brain activities especially about the comparison between the condition when the target item appeared at the center position of a string and the single‐element condition to reflect the VAS‐related processing with balancing the target position, which probably relied more on covert shifts instead of overt shifts. Bilateral SPLs have been suggested to play distinct roles in spatial attentional shifts and sustained attention, and especially the responses of RSPL were related to the overt shifts but not the covert shifts whereas LSPL exhibited significant activation in covert as well as overt attentional shifts (Vandenberghe, Gitelman, Parrish, & Mesulam, 2001). Since the visual 1‐back task in the present study required participants to focus on the screen center, and the data analyses controlled the possible influence of various target positions, and therefore the covert but not overt attention shift might be emphasized in the present study, with inducing the greater activation in LSPL while weakening the involvement of RSPL during the visual simultaneous processing.

In addition, although bilateral SPLs belonging to DAN have been found to be involved in visual spatial analysis (Cao et al., 2010), LSPL is more greatly associated with the visuospatial processing of the characters (Deng, Booth, Chou, Ding, & Peng, 2008; Deng, Guo, Ding, & Peng, 2012), while RSPL play a crucial role in basic visual analysis regarding spatial or nonspatial attention (Park et al., 2016). Previous studies on native speakers of alphabetic languages learning the second language such as Chinese and Japanese revealed that the reading acquisition of logographic languages would bring about neural plasticity in SPLs' functions. In detail, the experience regarding learning logographic language strengthened the activation in LSPL (Deng et al., 2008), and weakened the activation in RSPL (Sakai, Kuwamoto, Yagi, & Matsuya, 2021). Some researchers indicated that since Chinese characters have complex visual forms, and Chinese reading in daily life would exercise on the abilities of visual‐orthographic analysis, with resulting in more robust activities in LSPL (Deng et al., 2012; Kuo et al., 2004). Meanwhile, the experience of Chinese characters' processing would improve our basic spatial abilities, with saving our extraneous energy to attend to and to process the nonverbal visual stimuli (e.g., symbols), which may correspond to the decrease in right superior parietal activities (Sakai et al., 2021). Accordingly, it could be proposed that previous neuroimaging studies on VAS were all in the context of French, in which the reading experience of linear alphabetic scripts may not exert a significant influence on the brain function of SPLs. By contrast, the participants in the present study were all Chinese skilled readers, and their sufficient experience of Chinese characters would emphasize the role of LSPL. Moreover, the visual complexity of the current stimuli in VAS tasks seemed to be more simple than that of Chinese characters, with less involvement of RSPL.

Moreover, further position‐based analyses revealed that only responses of LSPL exhibited significant differences among positions, especially, greater activation was observed when the target appeared in the outer position (e.g., the first or fifth position) of a string as compared to the condition that the target was presented in the middle position. Consistently, analysis of behavioral data showed an effect of fixation advantage in the attentional distribution during multielement processing, which was in line with previous findings (Huang, Lorusso, Luo, & Zhao, 2019; Zhao et al., 2019; Ziegler et al., 2010). The combination of the present behavioral and neuroimaging findings may suggest a possible role of LSPL in distributing attentional resources regarding VAS. This left parietal region is critical in the division of visual attention over large segments of the visual field (Robertson, Lamb, & Knight, 1988), and it is a region implicated in spatial perception, attention, and working memory/short‐term memory (Foxe et al., 2016; Panichello & Buschman, 2021). Moreover, left intraparietal sulcus (including LSPL) has been found to be activated proportionally to demand on the top‐down visual spatial attention with cue‐induced orienting (Hahn, Ross, & Stein, 2006), and it has been suggested that controlled attentional processes were disrupted in patients with lesions centered in left posterior parietal cortex (Robertson et al., 1988). Accordingly, based on previous literature (Bosse et al., 2007; Bundesen, 1990; Bundesen, Vangkilde, & Petersen, 2015; Stefanac et al., 2019) and the current results of the behavioral and neural responses regarding the position effect, it could be proposed that during the simultaneous processing of the symbols within the window size of VAS, the distribution pattern of VAS resources was originally imbalanced with showing a middle‐position advantage. Therefore, further processing the item at outer positions required more top‐down attentional control as compared to that at the middle position, with evoking greater brain activities in LSPL.

### 4.2 Multiple roles of bilateral inferior frontal gyrus

Bilateral IFGs were significantly and consistently activated by VAS‐related processing. Previous studies (Fox et al., 2006) indicated that inferior frontal cortex belonging to ventral attention network has been found to be right‐lateralized. Accordingly, we further compared the inferior frontal activities between two hemispheres in each condition regarding target presence, and found greater activation in RIFG than that in LIFG in the target‐absent condition, which was consistent with the right‐lateralization in previous research (Fox et al., 2006); while no lateralization difference was observed in the target‐present condition. According to relevant literature (Corbetta et al., 2008; Wen, Yao, Liu, & Ding, 2012), it could be proposed that when the target item was presented in a string (i.e., target‐present condition), bilateral IFGs might play roles in stimulus‐driven bottom‐up attentional reorientation, that was, detecting all the symbols in one string instead of limiting to the attentional focus. Whereas, in the target‐absent condition, no item in one string was identical with the target stimulus and a rejective response was expected to be made, meanwhile, the function of RIFG in inhibitory control may be greatly induced as compared to LIFG with reference to relevant literature (Kolodny, Mevorach, & Shalev, 2017; Silva et al., 2019). Moreover, as shown in Figures 3 and 4, bilateral IFGs in our study seemed to locate in an overlapping region between VAN and DAN, which has also been found in previous studies (Fox et al., 2006; Vossel, Geng, & Fink, 2021). This special pattern suggested the role of IFGs in shifting attention by sending bottom‐up signals from VAN to DAN. From the above results, diversified roles of IFGs (especially RIFG) were observed, that was, IFGs participated not only in visual simultaneous processing but also in response inhibition.

### 4.3 Suppression on TPJs during multielement processing in the target‐absent condition

Bilateral TPJs, ROIs belonging to VAN, were only significantly activated in the target‐absent condition rather than the target‐present condition, suggesting brain activations in these regions may not be associated with VAS but with inhibition, which conflicted with previous findings reporting the involvement of TPJ in VAS‐related processing (Peyrin et al., 2011; Reilhac et al., 2013; Valdois et al., 2014). However, previous researches did not consider the conditions regarding the target presence, meanwhile relevant results may also be affected by linguistic processing of verbal stimuli (Peyrin et al., 2011; Reilhac et al., 2013; Valdois et al., 2014). In the target‐absent condition of the present study, bilateral TPJs were less activated in multielement processing of the nonverbal stimuli as compared to the single‐element processing, revealing the suppression on TPJs activities during rapid simultaneous processing, which was consistent with previous studies indicating that TPJs as “circuit breaker” interrupted ongoing processes by reorienting new stimuli (Parks & Madden, 2013) and was suppressed during detecting target from irrelevant information (Farrant & Uddin, 2015). A large number of distractor stimuli to be rejected in the multielement condition as compared to that in the single‐element condition provided more possibilities for activating TPJs to reorient. Therefore, it could be inferred that to ensure the success of target detection and discrimination during VAS processing, TPJs should be greatly suppressed especially in the multielement condition. However, the lack of significant temporoparietal activities in the target‐present condition may suggest that the recruitments of TPJs did not stably reflect its role in VAS processing but possibly in inhibition processes.

## 5 INVOLVEMENT OF DORSAL AND VENTRAL ATTENTION NETWORKS IN VAS PROCESSING

The current result of more robust activation in ROIs of DAN as compared to that of VAN in VAS‐related processing was consistent with the results of previous studies (Lobier et al., 2012, 2014). Since the close relationship between top‐down (or endogenous) attention and DAN (Berndt et al., 2019; Corbetta & Shulman, 2002), the present finding was generally consistent with the study of Valdois et al. (2019), revealing that VAS skills greatly relate to endogenous attention as compared to exogenous attention. The flexible‐attention framework proposed by Zhang and Kay (2020) provided a possible explanation for this finding. The framework indicated that as visual stimuli appearing far away from the center position (i.e., weak stimuli) with demand for processing the weak stimuli, bottom‐up stimulus‐driven response (e.g., neural responses in VAN) declined but top‐down attentional modulation (e.g., neural activities in DAN) increased (Zhang & Kay, 2020). In the present study, the contrasts of activation between the multielement condition and single‐element conditions mainly reflected the rapid visual simultaneous processing, especially for processing stimuli in the noncenter positions. Consequently, participants should accumulate enough sensory evidence to make a perceptual decision on the weak stimuli (i.e., stimuli in the noncenter positions), in which the top‐down goal‐directed attention (i.e., DAN) would be disproportionally enhanced while the activities relating to the physical salience (i.e., VAN) would be suppressed (Farrant & Uddin, 2015; Jimenez et al., 2016) for the accumulation in these weak neural responses constituting weak sensory evidence.

The remarkable activation in regions belonging to DAN may also be associated with the property settings of the current task. Different from the traditional visual 1‐back task in which the string was firstly presented and followed by a target of a single letter or symbol (i.e., a post cue), the target symbol (i.e., a pre cue) was changed to be presented before each session in the current study to make the task more suitable to fMRI research. In this prospective task, participants were required to search the target (pre‐cue) within each symbol string. This procedure might greatly rely on the top‐down attentional control (Panichello & Buschman, 2021). Since top‐down task‐driven searching has been suggested to recruit the activities of brain regions relating to DAN (Ekstrand et al., 2019; Hahn et al., 2006), and thus this task setting may partially explain the great involvement of DAN in the current VAS task. However, a recent neural study on rhesus monkeys (Panichello & Buschman, 2021) reported that parietal and prefrontal activities exhibited similar patterns between the post‐cue and pre‐cue tasks. Moreover, because the trials of different conditions about target presence and target position were randomly presented, participants should simultaneously process the whole string regardless of whether the target was presented before or after the string. In addition, if the involvement of DAN was mainly related to visual search, and then it could be expected that there was greater activation in the target‐present condition than that in the target‐absent condition. In the current study, activation in LITG conforms to this hypothesis, which may reflect the role of LITG in attentional selection. But beyond that, the present findings showed that the two conditions of target presence did not differ from each other in intensities of DAN‐related activities, and these results support the rationality and validity of the current task that reflected the VAS‐related processing.

Besides, although a single‐element identification task was set as a baseline to decrease the possible interruptions from memory factors, the present task was implicated in working memory and short‐term memory to some extent. Previous researches reported that memory‐guided visuospatial attention recruited DAN (Rosen, Stern, Devaney, & Somers, 2018), and the intraparietal sulcus (overlapping with SPL in the present study) was obviously connected with anterior frontoparietal areas in the contrasts between visual short‐term memory and visual attention (Panichello & Buschman, 2021; Sheremata, Somers, & Shomstein, 2018). Therefore the present results reporting significant activation of DAN may also reflect the memory‐guided attention in the VAS task. However, some researchers indicated that there were several underlying cognitive processes contributing to the VAS according to the theory of visual attention (Bundensen, 1990), including the visual short‐term memory storage (Bogon et al., 2014; Dubois et al., 2010; Stefanac et al., 2019). Accordingly, the memory factor may be one of the subcomponents regarding VAS. Future studies could further explore the VAS‐related mechanism about maintaining the multielements in short‐term memory after simultaneously decoding the string.

## 6 CONCLUSIONS

The present study provides new insights into the neural mechanism of VAS via a modified visual 1‐back task with nonverbal stimuli and nonverbal responses, considering the possible influences of target presence and target position. Current results reveal that both DAN (i.e., LSPL) and VAN (i.e., bilateral IFGs) are neural markers of VAS‐related processing. In particular, greater activation reported in LSPL as compared to that in bilateral IFGs, suggests a greater involvement of DAN in rapid visual simultaneous processing. Moreover, diversified roles of IFGs (especially RIFG) are found in both VAS‐related processing and inhibition control. Besides, TPJs, classical regions of VAN, are only activated in the target‐absent condition but not in the target‐present condition, revealing that TPJs might mainly function in inhibition processes instead of in visual simultaneous decoding of multiple elements regarding VAS. The current findings bring some enlightenments for further exploration of the functional connectivity across these critical regions, and the neural correlates of the relationship between VAS and reading (especially, preliminary analysis of the relation between the VAS‐related brain activities and Chinese reading was in S1 section of the Supporting Information). Meanwhile the present study indicates possible candidates for future studies to investigate the atypicality of attentional disorders and reading disabilities.

## CONFLICT OF INTEREST

The authors declare that they have no conflict of interest.

## AUTHOR CONTRIBUTIONS

*Jing Zhao*: supervised the project, designed the whole study, analyzed the data, wrote and revised the Introduction and Discussion sections of the manuscript. *Junkai Wang*: analyzed the data, prepared the figures, wrote and revised the Method and Result sections of the manuscript. *Chen Huang*: programmed the visual attention experiment, collected the neuroimaging data, analyzed the data, wrote and revised the Method and Result sections of the manuscript. *Peipeng Liang*: supervised the project, designed the visual attention experiment, analyzed the neuroimaging data, edited and revised all the parts of the manuscript.

## ETHICS APPROVAL STATEMENT

The research was approved by the Research Ethics Committee of School of Psychology, Capital Normal University. This study was carried out in accordance with The Code of Ethics of the World Medical Association (Declaration of Helsinki) for experiments involving humans.

## Supporting information


**Appendix** **S1**: Supporting information

## Data Availability

The datasets in this study are available on request to the corresponding author.
