# Supplementary material for: Involvement of the dorsal and ventral attention networks in visual attention span
Source: Hum Brain Mapp. 2022 Jan 4;43(6):1941–54. doi: 10.1002/hbm.25765 (PMC8933248; doi:10.1002/hbm.25765)
Supplement: Supplementary file 1 — Appendix S1: Supporting information [file HBM-43-1941-s001.docx]

**Supporting Information**

**S1. Relationship between VAS-related brain activations and Chinese reading**

In order to confirm which region(s) is(are) functionally related to the role of visual attention span in reading, we further examine the possible relationship between VAS-related brain activations and Chinese reading. Since reading procedure mainly involves visual-to-phonological mapping and visual-to-semantic mapping, and we utilized oral and silent reading tests to separately reflect these two types of linguistic processes. In details, the main goal in oral reading is to pronounce every word/character with understanding being secondary, which mostly depend on the visual-to-phonological mapping; in contrast, the main goal in silent reading is to comprehend and assimilate the meaning of the text, which mainly involves in the visual-to-semantic decoding (van den Boer, et al., 2014; Wang et al., 2015).

**S1.1 Chinese reading tests**

Sentence reading tasks (van den Boer et al., 2014; Zhao et al., 2019) were used to examine Chinese reading skills separately in oral reading mode and silent reading mode.

Each participant received both modes of the reading tests, in which the order of the two reading tests were balanced. The interval between the tests in two reading modes was at least one hour to reduce the possible influence of practice effect. The split-half reliability was 0.85. This was a computerized test, which was programmed and presented by E-prime 1.0. There were 25 true sentences and 25 false sentences in the formal test. The sentences were all about simple facts (e.g., “一个星期有七天” means that “There are 7 days in a week”). Within each trial, the target of a complete sentence first appeared. The participants were instructed to orally or silently read the sentence as quickly and accurately as possible, and then to press the space bar once they had finished reading the sentence. The interval between the onset of the sentence and the time of pressing the space bar was recorded as the reading time. A screen for sentence verification was further presented to ensure the validity of the above sentence reading. The participants were required to press different keys to determine the accuracy of the sentence. The judgment accuracy was recorded, which was higher than 90% for all the adult participants and thus was not put into further data analyses. The reading speed of accurately determined sentences was calculated as the ratio of the sentence length to the corresponding reading time (c/min), which was submitted to the following analyses.

**S1.2 Results and discussion about the relationship between VAS-related brain activations and Chinese reading**

Partial correlation analyses were conducted to examine the relationship between VAS-related activations (especially in LSPL and bilateral IFGs) and sentence reading skills while the participants’ age were controlled. In detail, VAS-related activations were restricted to brain activities in target-present condition (i.e. the comparison of target-present trials between multi-element identification when the target appeared at the centre position of a string and the single-element identification), as well as brain activities regarding the position effect (i.e. the comparison of the target-present trials in the multi-element session between the outer and centre positions of a string).

Results (Table S1) showed that activation in LSPL was correlated with silent reading especially in the position effect rather than in the target-present condition. Particularly, the greater activations in LSPL when the target appeared in the outer vs centre positions were, the higher reading speeds were. Moreover, brain activities in bilateral IFGs were correlated with oral reading speed. That is, greater activations in IFGs in the condition when the target appeared at the central position of a string as compared to that in the conditions of single-element session corresponded to the higher reading speed.

**Table S1 Correlation coefficients in the relationship between VAS-related activations and Chinese reading**

| **ROIs** | | **Oral reading speed** | **Silent reading speed** |
| --- | --- | --- | --- |
| ***Target-present condition*** | | | |
| **LSPL** | Confirmatory | *r*=-0.18, *p*=0.37 | *r*=0.12, *p*=0.56 |
|  | Exploratory | *r*=-0.30, *p*=0.14 | *r*=0.04, *p*=0.86 |
| **LIFG** | Confirmatory | ***r*=0.41*, *p*=0.04** | *r*=-0.16, *p*=0.43 |
|  | Exploratory | *r*=0.30, *p*=0.15 | *r*=-0.07, *p*=0.73 |
| **RIFG** | Confirmatory | ***r*=0.37^+^, *p*=0.06** | *r*=0.26, *p*=0.20 |
|  | Exploratory | *r*=0.22, *p*=0.29 | *r*=-0.17, *p*=0.40 |
| ***Position effect*** | | | |
| **LSPL** | Confirmatory | *r*=-0.30, *p*=0.15 | ***r*=-0.42*, *p*=0.04** |
|  | Exploratory | *r*=-0.28, *p*=0.19 | ***r*=-0.45*, *p*=0.03** |

Note. LSPL, left superior parietal lobule; LIFG, left inferior frontal gyrus; RIFG, right inferior frontal gyrus. Confirmatory, confirmatory analysis; Exploratory, exploratory analysis. *, p<0.05; +, p<0.1.

Previous behavioral research has found that visual attention span capacity was closely related to both of the oral and silent sentence reading in Chinese skilled readers (Huang et al., 2019). The present results revealed the neural mechanisms for the VAS-reading relationship, with further suggesting the dissociation of the mechanisms between the “VAS-silent reading” relation and the “VAS-oral reading” relation. In details, VAS-related activations in bilateral IFGs (belonging to ventral attention network) were associated with oral sentence reading, while VAS-related activations in LSPL (belonging to dorsal attention network) were associated with silent sentence reading.

When skilled readers silently read Chinese scripts, they mainly depend on the direct connections between the visual forms and the corresponding meanings of Chinese characters (Siok & Fletcher, 2001). Posterior parietal cortex has been reported to be recruited to form a memory-attention network which integrate memory/experience and stimulus-based representations to guide attention (Rosen et al., 2018). Accordingly, it could be proposed that Chinese reading experience may play a role in guiding the attentional distribution through a top-down method which recruits the involvement of SPL. As to the oral reading mode, the skilled readers have to transform the orthography to phonology to achieve the oral reading performance, in which the detailed features and properties of each characters in a visual attention window would be rapidly decoded via a bottom-up way to finally retrieve their correct pronunciations, which involves the activation in VAN-related areas and is consistent with previous findings reporting the role of VAN in globally lexical route (Ekstrand et al., 2019).

**Reference**

Ekstrand, C., Neudorf, J., Kress, S., & Borowsky, R. (2019). How words and space collide: lexical and sublexical reading are reliant on separable reflexive and voluntary attention regions in hybrid tasks. *Cortex*, 121, 104-116.

Huang, C., Lorusso, M. L., Luo, Z., & Zhao, J. (2019). Developmental differences in the relationship between visual attention span and Chinese reading fluency. *Frontiers in Psychology*, 10, 2450

Rosen, M.L., Stern, C.E., Devaney, K., & Somers, D.C. (2018). Cortical and subcortical contributions to long-term memory-guided visuospatial attention. *Cerebral Cortex*, 28, 2935-2947.

Siok, W. T., & Fletcher, P. (2001). The role of phonological awareness and visual- orthographic skills in Chinese reading acquisition. *Developmental Psychology*, 37, 886–899.

van den Boer, M., van Bergen, E., & de Jong, P. F. (2014). Underlying skills of oral and silent reading. *Journal of Experimental Child Psychology*, 128, 138–151.

Wang, X., Yang, J., Yang, J., Mencl, W. E., Shu, H., & Zevin, J. D. (2015). Language diﬀerences in the brain network for reading in naturalistic story reading and lexical decision. *PLoS One*, 10, e0124388

**S2 Laterality effects in brain activations regarding visual attention span**


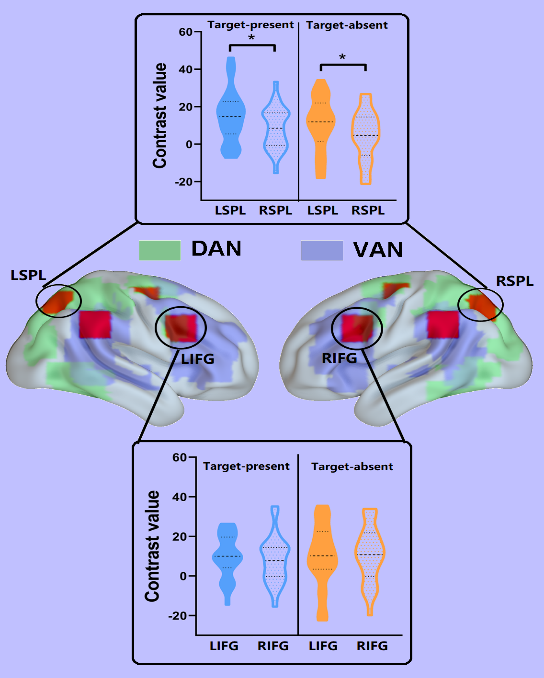


**Figure S1 Laterality effects in regions of interest including SPLs and IFGs in the confirmatory analysis.** *, *p*<0.05.
